# *Ginkgo biloba* leaf extract EGb 761^®^ as a paragon of the product by process concept

**DOI:** 10.3389/fphar.2022.1007746

**Published:** 2022-10-11

**Authors:** Žarko Kulić, Martin D. Lehner, Gunnar P. H. Dietz

**Affiliations:** ^1^ Preclinical Research and Development, Dr. Willmar Schwabe GmbH and Co., KG, Karlsruhe, Germany; ^2^ Global Medical Affairs, Dr. Willmar Schwabe GmbH and Co., KG, Karlsruhe, Germany; ^3^ University Medical Center, Göttingen, Germany

**Keywords:** phytopharmacology, adulteration, purity, extraction process, ginkgolides, proanthocyanidins, flavonoids, bilobalide

## Abstract

It is an often-neglected fact that extracts derived from the very same plant can differ significantly in their phytochemical composition, and thus also in their pharmacokinetic and pharmacodynamic properties which are the basis for their clinical efficacy and safety. The *Ginkgo biloba* L. [*Ginkgoaceae*] special extract EGb 761^®^ is one of the best-studied plant extracts in the world. In the present review, using that extract as a paradigm, we describe insights how climate, the harvest region, processing of the plant material, the drying process, the extraction solvents, and the details of the subsequent process steps substantially impact the quality and uniformity of the final extract. We highlight the importance of regulating active constituent levels and consistent reduction of undesired substances in herbal extracts. This is accomplished by a controlled production process and corresponding analytical specifications. In conclusion, since extracts derived from the same plant can have very different phytochemical compositions, results from pharmacological, toxicological and clinical studies gained with one specific extract cannot be extrapolated to other extracts that were generated using different production processes. We propose that the heterogenous nature of extracts should be meticulously considered when evaluating the efficacy and safety of plant-derived remedies.

## Introduction: Every plant is special, every extract is a maverick

Plants can be considered the “best organic chemists” on our planet. While “only” less than 200 million compounds have been registered to date (CAS REGISTRY | CAS), that number is likely far exceeded by the number of compounds estimated to occur in nature. This can be attributed to over 3.5 billion years of evolution, that allowed those compounds to be optimized depending on the respective environmental requirements. To survive on land, higher plants had to construct a mechanical structure that responds to terrestrial gravity, and had to synthesize chemical substances to protect the cells against reactive oxygen species, that may, for instance, be generated by solar radiation. Antioxidant activity is a key process in response to various environmental stresses such as light and high temperature. Most of the natural plant metabolic processes could not be reproduced by chemic-synthetic methodology.

Metabolites like carbohydrates, amino acids, nucleotides and fatty acids serve as the primary source of energy and biomass of higher organisms. Metabolites like polyphenols, terpenes, alkaloids, to name only the most prominent ones, which are produced by the plant in response to interaction with its environment, often bear pharmacological effects on humans and animals. This may be due to the long-term interaction and co-evolution of living organisms with each other. Man-made chemical compound libraries, on the other hand, as large as they may be in our days, are the result of a relatively limited toolbox of manual organic synthesis. Most molecules in such collections are usually not at all apt for interaction with structures occurring in nature. The chemical space they cover is thus quite limited in comparison to the chemical space explored by nature, with, e.g., aconitine being a “structural masterpiece.” For many natural compounds, it has not been possible to date to produce them economically in relevant quantities. For instance, although organic chemists accomplished the synthesis of Ginkgolides, those processes are far from being cost-effective (Schäfer, 2015). Therefore, a large share of today’s synthetic drugs is structurally derived from plant secondary metabolites (Newman and Cragg, 2020). Medicinal plants have a widespread coverage and tradition of use throughout almost all human cultures (Petrovska, 2012) and are also actively applied by animals (Shurkin, 2014).

While the direct consumption of medicinal plants is common for aromatic spices like garlic, fennel, thyme, turmeric, etc. the major share of medicinal plants is refined before consumption to enrich the therapeutic components and, in some cases, deplete unwanted components. The refinement methods span from simple hot water infusions like tea, maceration, and percolation, to complex multistep extraction processes like the ones known for extracts of *G. biloba* leaves. Although refined, the obtained material is very often still a highly complex mixture consisting of up to thousands of different compounds in varying concentrations. The yield and the phytochemical composition can vary significantly even between two extracts from the same plant species. This is intuitive with one of the most straightforward extractions known: infusions of tea [*Camellia sinensis* (L.) Kuntze (*Theaceae*)] leaves. Its antioxidant capacity and total phenolic and catechin content (Kyle et al., 2007), the stimulating caffeine (Ramalho et al., 2013), and the relaxing theanine (Boros et al., 2016) concentrations increase with prolonged infusion time. Moreover, the cultivar variety, growing environment, manufacturing conditions, and grade (particle size) of the tea leaves, the use of tea bags, including their size and material, the amount of tea and water used, infusion time, and agitation, are all major determinants of the component concentrations of tea beverages (Astill et al., 2001). This is equally evident for wine made in different years, or from different regions of the world, even when the same grape variety was used for production. Differences in the outcome can be even more dramatic for plant extracts used for herbal medicinal products. Parameters like plant particle size, extraction solvent, duration, temperature, mixing, drug to solvent ratio, drug to extract ratio (DER), etc. determine the phytochemical composition of the obtained extract, with profound consequences on the efficacy and tolerability of the final product. This principle is evident regarding medicinal plants with widespread use like, e.g., aerial parts of common sage [*Salvia officinalis* L. (Lamiaceae)]. European authorities acknowledge traditional use in four different indications (Committee on Herbal Medicinal Products (HMPC), 2016). However, the preparations are in part different for the respective indications. While a liquid extract prepared with 50% (v/v) ethanol and a DER of 4–5:1 can be used against excessive sweating, it can’t be used for the relief of oropharyngeal inflammations. However, the latter indication can be treated with a liquid extract prepared with 70% (v/v) ethanol and a DER of 1:1. Another example of this principle concerning the tolerability is black cohosh [*Actaea racemosa* L. (Ranunculaceae)]. European authorities, acknowledge the well-established use against menopausal complaints for three different preparations (Committee on Herbal Medicinal Products (HMPC), 2018). However, the maximum daily dose is different for each one. While, e.g., a daily dose of 6.5 mg is allowed for the dry extract prepared with 60% (v/v) ethanol and a DER of 4.5–8.5:1, only 5.0 mg is allowed for the dry extract prepared with 40% (v/v) isopropanol and a DER of 6–11:1. These examples illustrate the aforementioned phytochemical differences of two extracts prepared by a different protocol.

For production on a large scale, different manufacturers employ facilities and equipment of disparate size, energy consumption, automation grade, and production protocols. Here, the multidimensionality of all these parameters boil down to a concept which is referred to as product-by-process and which is essential for the final quality of the product. This means that an extract of one plant species can differ substantially in its composition among manufacturers. Some manufacturing processes with their respective products are patented, which reflects the unique quality of the respective extracts.

Extracts of the leaves of *G. biloba* are used therapeutically for different indications, depending on the regulatory and geographic context. Whereas cardiovascular diseases and cerebrovascular disorders are depicted in the Chinese Pharmacopeia for Ginkgo leaf tablets, the European Herbal Monograph describes the use of *G. biloba* leaf extract-based herbal medicinal products for the improvement of (age-associated) cognitive impairment and of quality of life in mild dementia. In addition, powdered *G. biloba* leaf is traditionally used for the relief of heaviness of legs and the sensation of cold hands and feet associated with minor circulatory disorders (Committee on herbal medicinal products (HMPC), 2015).

EGb 761^®^ is a special extract generated from dried *G. biloba* leaves, that will be described in detail in this review. Medicinal products containing EGb 761^®^ as active substance have shown to be effective in tinnitus (von Boetticher, 2011), vertigo (Hamann, 2007), as well as cognitive impairment and dementia (Gauthier and Schlaefke, 2014; Tan et al., 2015; von Gunten et al., 2016; Savaskan et al., 2017). Recent preliminary data suggests that EGb 761^®^ might alleviate post-COVID-19 cognitive deficits (Zifko et al., 2022).

The potential phytochemical differences between extracts from different manufacturers will be discussed in the following review, with specific focus on the special extract EGb 761^®^ as the archetype for the product-by-process concept.

## It starts with the beginning: Even cutting trees affects the later product

The first major factor of the final quality of an extract is the plant material. Seasonal changes in leaf development are influenced by the latitude and climate of the cultivation area. Amounts of plant secondary metabolites are heavily dependent on environmental factors such as light, temperature, precipitation, soil fertility and salinity (Yang et al., 2018). In addition, cultivation techniques and use of abiotic stress signals strongly determine the phytochemical composition (Ramakrishna and Ravishankar, 2011).

For *G. biloba*, the plantations and sites of harvest are scattered around the globe. The optimal conditions for the *G. biloba* plants are subtropical climates with sandy and well drained soils with pH values between 5—6 (Schmid and Balz, 2005). The histological characteristics of *G. biloba* leaves are known to adjust to different climate conditions (Sun et al., 2003).

Planting Ginkgo seedlings at a moderate density away from one another, as compared to low or high density, provides a good leaf yield per farmland area concomitantly with an increased flavonoid, ginkgolide and bilobalide contents (Lu et al., 2021).

The chemical composition and thus the starting raw material for the final product is impacted by differences in soil quality and climate parameters like precipitation/water access (Hao et al., 2007; Wang et al., 2013; Wang et al., 2014), light intensity, temperature (Wang et al., 2013; Wang et al., 2014), UV radiation (Zhao et al., 2020), salinity (Xu et al., 2020), but also the plant and leaf age and time of harvest (Chen et al., 2006; Goto and Usuki, 2012; Horbowicz et al., 2021a; Horbowicz et al., 2021b). The known genomic variability of *G. biloba* trees (Šmarda et al., 2018) may also influence the constituent contents of the leaf material.

Over the last decades, much knowledge has accumulated how specific measures of *G. biloba* farmers influence the phytochemicals in the leaf, which is exemplified by recent studies indicating that truncation down to the base of the tree can rejuvenate the plant and increase biomass and flavonoid content (Lu et al., 2022). The weed control, watering, and the harvesting time and method also play an important role. Reliable plantation management and well-trained collectors support the quality by applying Good Agricultural and Collecting Practice (GACP) regulations. Ni et al. (2017) reported that after harvest but prior to extraction, the treatment of *G. biloba* leaves with NaCl or UV-B light increases flavonoid levels. Moreover, it cannot be excluded that other post-harvest processing of the leaves like drying, packaging, transport and storage might impact the phytochemical composition by, e.g., the hydrolysis rate of flavonol glycosides and terpene trilactones (van Beek and Montoro, 2009), Therefore these process steps are covered by GACP regulations as well in order to ensure a consistent quality of the herbal starting material after harvesting.

**FIGURE 1:**
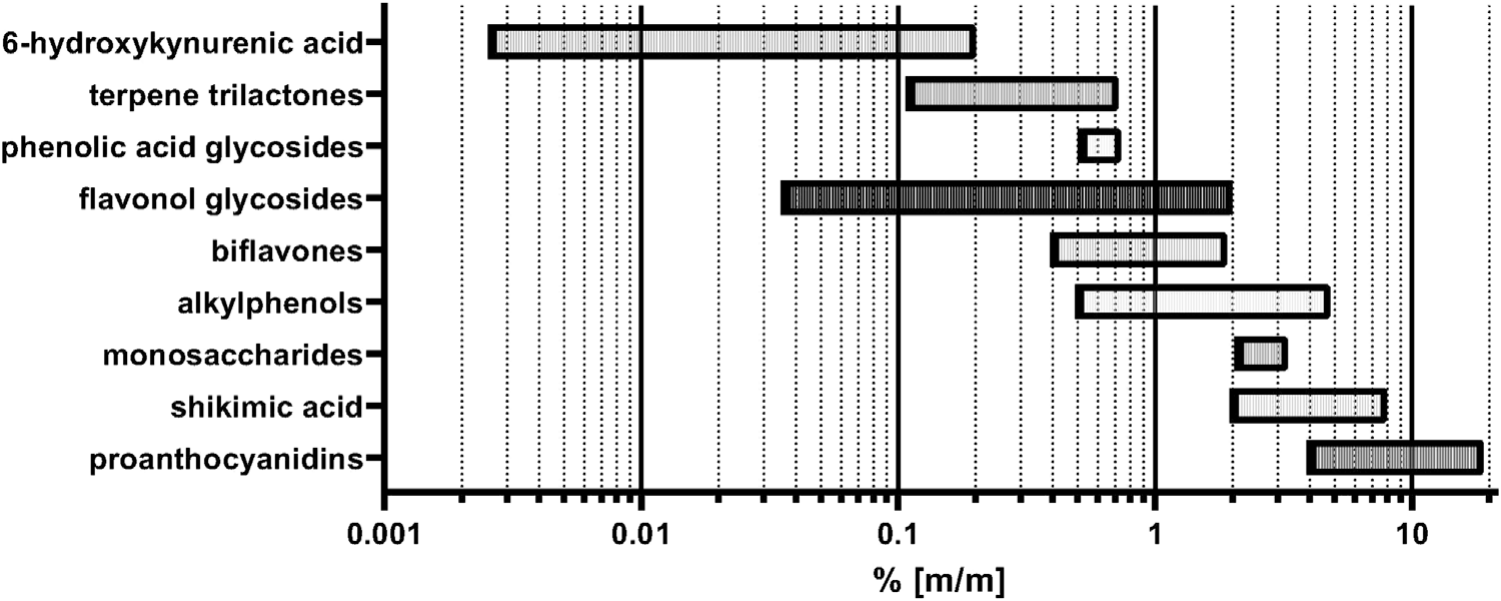
Summary of minimum and maximum concentrations of constituents in *G. biloba* leaves according to literature and in cases of the flavonol glycosides and PACs own data on upper limits.

The importance of the above-mentioned agricultural factors and harvesting details for the quality of the raw material becomes evident by analytical comparisons. Variations of many constituent levels in the leaves have recently been reviewed (Gafner, 2022). Here, we reprocess the main information and provide additional details and viewpoints on this subject. The levels of two compound classes used for standardization of the finished products, namely flavonol glycosides and terpene trilactones vary between 0.036%—1.87% and 0.11%–0.72% in the leaf, respectively (Yao et al., 2012; Zhou et al., 2018; Lin et al., 2020; Wu et al., 2021). Our own analyses show even higher flavonol glycoside contents of up to 2% (unpublished results). The age of the trees and the time of harvest of the leaves also play an important role for the concentration of these two groups of compounds. The total content of flavonol glycosides and terpene trilactones in the leaves was found to decrease with progressing age of the tree. Moreover, there are seasonal variations: Total flavonol glycosides amount peaks in May, while the amount of terpene lactones is highest in August and September (Ding et al., 2015). Also the leaf levels of compounds which are unwanted in the finished products, namely biflavones and alkylphenols differ in their content between 0.4%—1.9% (Spieß et al., 2011; Wu et al., 2021) and 0.5%–4.8%, respectively (Choi et al., 2004; van Beek and Montoro, 2009; Zhou et al., 2017). Apart from these compounds which are usually standardized in the finished products, there are also compound classes which are not standardized, the content of which also varies in the finished products due to different production processes and different raw materials used by manufacturers. For these compounds like proanthocyanidins (PAC), 6-hydroxykynurenic acid (6-HKA) and shikimic acid, content levels in the leaf range between 4%—12%, 0.0003%–0.2%, and 2%—8%, respectively, with some variation between the different literature sources (Qa’dan et al., 2010; van Beek and Montoro, 2009; Cao et al., 2018b; Yao et al., 2017; Spieß et al., 2011). Our own data demonstrate even higher contents of PACs >19% for some harvest regions. The content of phenolic acid glycosides was found to vary from 0.515%—0.735% in different regions of harvest (Li et al., 2020). Monosaccharide content was also found to vary between 2.09%—3.27% in the leaves (Wei et al., 2018). The aforementioned phytochemical concentration ranges are summarized in Table 1 and Figure 1. To simultaneously monitor the content of most of the phenolic and terpenic substances in the plant material, a LC-MS multi reaction monitoring (MRM) based method was developed, also showing great differences in the analyte concentrations depending on the region of harvest (Yao et al., 2013). Also, other LC-MS based methods were published for simultaneous quantitation of multiple components (Wang et al., 2017; Zhao et al., 2017; Wang et al., 2019).

**TABLE 1:** Summary of min/max. concentrations of constituents in *G. biloba* leaves according to current literature data and in cases of the flavonol glycosides and PACs own data on upper limits.

Constituent	Min content in % (m/m)	Max content in % (m/m)
flavonol glycosides	0.036	>2%
terpene trilactones	0.11	0.72
biflavones	0.4	1.9
alkylphenols	0.5	4.8
proanthocyanidins	4	19
shikimic acid	2	8
6-hydroxykynurenic acid	0.0026	0.2
phenolic acid glycosides	0.515	0.735
monosaccharides	2.09	3.27

Another quality issue is purity of the plant material. The high manufacturing cost of standardized *G. biloba* extracts tempts individual manufacturers to blend their preparations with other, cheaper material. Modern analytical methods revealed that the concentrations of, e.g., individual flavone glycosides not only differ substantially between products, but that in two out of five samples examined, admixture of flavone glycosides from cheaper sources was assumed (Ding et al., 2006). Plant material is frequently adulterated with other plants rich in flavonol glycosides like *Styphnolobium japonicum* (L) SCHOTT [*Fabaceae*] or Fagopyrum esculentum MOENCH [*Polygonaceae*] (Gafner, 2018). In between whole or cut *G. biloba* leaves, adulterants can be spotted by macroscopic pharmacognostic analysis. However, if powdered plant material is present, adulteration can only be detected by chemical analytic methods after extraction. (Hyphenated) HPLC methods for detection of isoflavones as adulteration markers proved to be robust (Harnly et al., 2012; Wohlmuth et al., 2014; Avula et al., 2015; Govindaraghavan, 2018; Bampali et al., 2021). Also, a DNA based analysis was developed for probing on *Sophora japonica* syn. Styphnolobium japonicum adulteration (Liu et al., 2018).

For manufacturing the proprietary extract EGb 761^®^, plant material from three different continents is used: Asia (China), North America (United States), and Europe (France) (Schmid and Balz, 2005). In the US (North Carolina) and France (Département Gironde) the plants are grown on controlled plantations. Plant material from China is sourced *via* long-term trading relations with contract cultivation sites and local farmers. Mechanical, flame-based or manual weed control throughout the year ensures high quality plant material devoid of pesticides and adulterants. Pruning of the trees ensures feasible harvesting, regular rejuvenation and high leaf production (Schmid and Balz, 2005). Harvest of the green foliage starts when both the optimum content of constituents is reached (Gafner, 2018), and the abscission zone of the deciduous *G. biloba* leaf has already somewhat weakened, which, depending on the environmental conditions, falls into July, August and September/October in the US, China and France, respectively. While the harvest in the US and France is carried out mechanically by modified cotton pickers, the material from Asia is harvested manually (Schmid and Balz, 2005). The drying of the fresh leaves is controlled by a steady process in drum dryers, yielding approximately 75% weight loss (Gafner, 2018) and long term storable herbal material. All aforementioned steps yield plant material of the best possible quality.

## Extraction process

There are numerous processes for the extraction of *G. biloba* leaves, with 1,100 patents being indexed by the CAS by May 2022 (search terms: Ginkgo and “extract” and “process”). Even more processes may exist, which are not (yet) patented. This compelling number is attributed to the phytochemical composition of *G. biloba* leaves containing both, beneficial and potentially harmful substances as stated above. The bulk of processes aims at enriching flavonol glycosides, terpene trilactones or other beneficial constituents while depleting ginkgolic acids in the final product (Figure 3). For that purpose, the primary liquid extract needs subsequent refinement steps splitting the phytochemicals into (multiple) waste streams and desired product, respectively. Current strategies to remove ginkgolic acids have been recently reviewed (Boateng, 2022). Among the manifold of published process protocols, the major mass of commercially relevant products is produced by refinement methods based on three chemical principles which are easily scalable. These principles include resin adsorption and desorption, precipitation and liquid-liquid extraction to get rid of ginkgolic acids, while retaining flavonol glycosides and terpene trilactones. However, the implementation details of these process protocols vary substantially. Covering details of all published process patents and scientific articles would be beyond the scope of this publication. Therefore, we focus on *G. biloba* extracts used as herbal medicine and covered by the major pharmacopoeias. Although compliance with pharmacopoeias is already the highest grade of regulation for *G. biloba* extracts, the processes allowed according to the pharmacopoeias may still vary to a large extent. This starts already in the first step: the primary extraction. While the European pharmacopoeia (Ph. Eur.) only allows primary extraction with a defined solvent ratio of 6:4 acetone:water (m/m) (European Directorate for the Quality of Medicines & HealthCare, 2020), the Chinese Pharmacopoeia (ChP) allows only hydroethanolic primary extraction without any specification of solvent ratios (Chinese Pharmacopoeia Commission, 2015). In fact, different concentrations of ethanol are used by different manufacturers, ranging from 30% up to 95% ethanol, as recently reviewed (Siting et al., 2022). The US Pharmacopoeia (USP) is even more tolerant on extraction solvents (“acetone-water mixture or other suitable solvents”) as long as the final specification on flavonol glycosides, terpene trilactones and ginkgolic acids is fulfilled (United States Pharmacopeial Convention, 1979 ff.) The interplay of the respective organic solvent with different concentrations of water and under different temperatures defines the solubility of *G. biloba* leaf constituents, and thus, the phytochemical composition of the primary extract. Also, the subsequent process steps are of major importance for the composition of the final product. These subsequent process steps are only specified in the ChP as application to a resin and subsequent elution, albeit without specifying the resin chemical selectivity or the elution details like solvent concentrations. This results in different resins being applied by different manufacturers (Siting et al., 2022). In contrast, the Ph. Eur. And the USP do not specify any process steps following the primary extraction, leaving the process design up to the manufacturers, as long as the final specification on constituents and the drug to extract ratio is met. It goes without saying that the difference of the primary extraction solvents as well as difference in all subsequent process steps largely impact the concentrations of the extracted phytochemicals in the final product.

One major flaw of all published extraction processes is the limited and incomplete definition of the final product, which is also discussed in more detail in chapter *The result generated by the process below*. In the final product, approximately 30% of constituents are specified, with all processes tuned towards these requirements. However, this leaves the residual 70% being prone to significant variation due to the multidimensional design of the variable process protocols. This means that the phytochemical composition apart from the specified compounds largely depends on the applied process in the final product. This principle is referred to as product-by-process, which is discussed in the following using the production process of EGb 761^®^ as a prime example.

**FIGURE 2:**
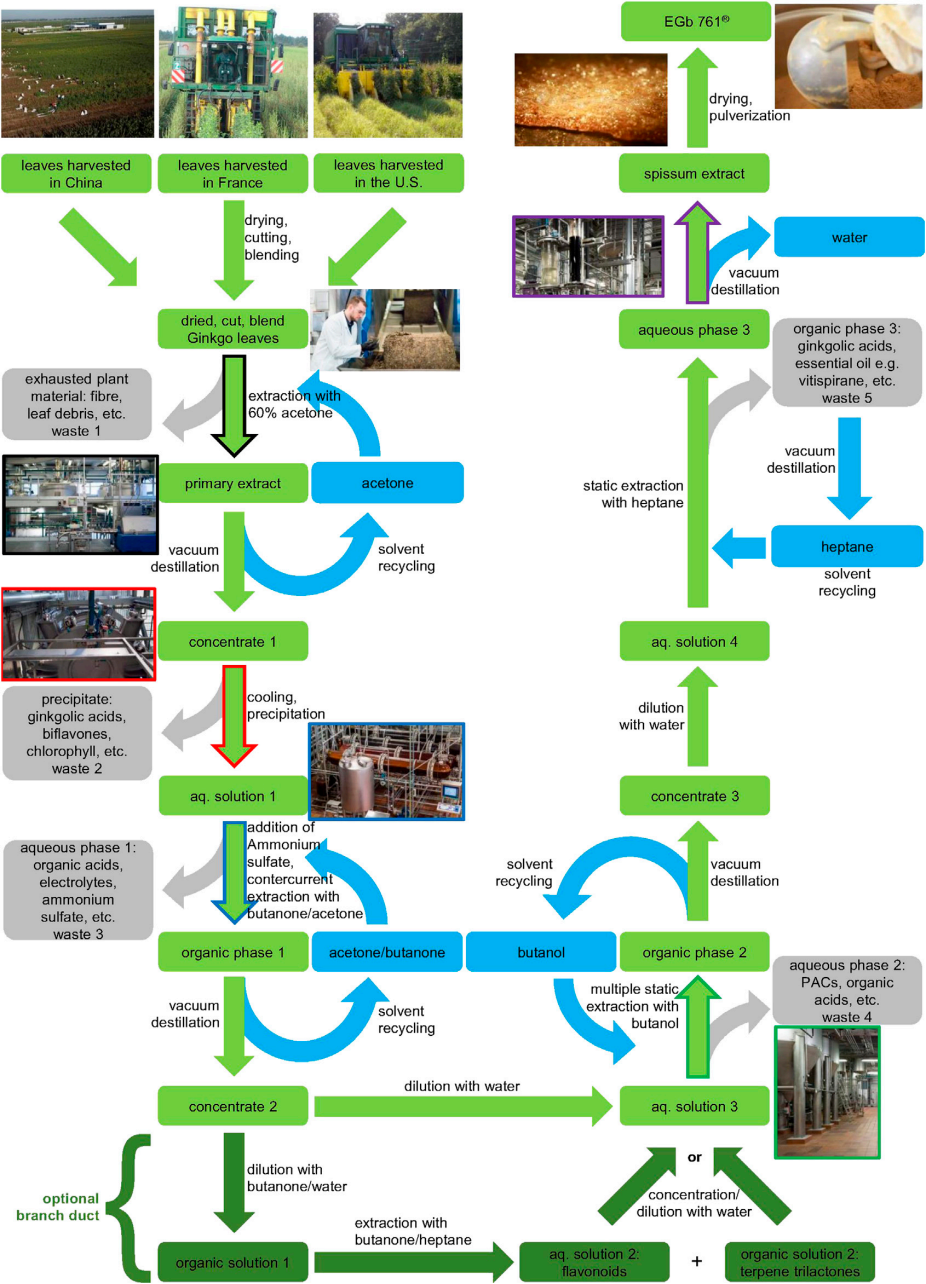
Scheme of the EGb 761^®^ multistep production process. Color coding: green–value phase, olive-green–optional branch duct, gray–waste phase, blue–solvents, fringe colors indicate the corresponding process facility units. The process is vastly different from other production processes (see Figure 4).

**FIGURE 3:**
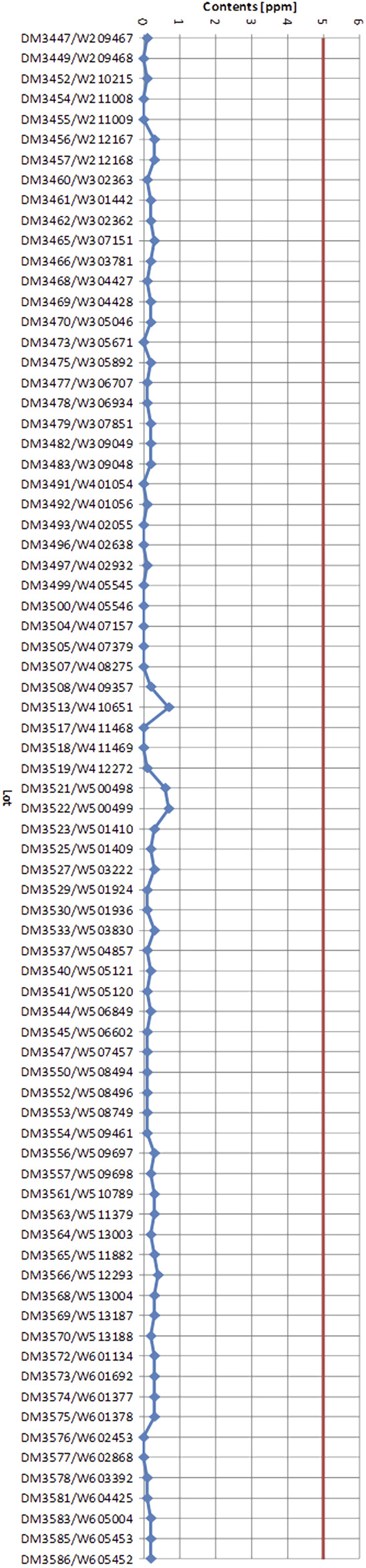
Ginkgolic acid contents of 75 EGb 761^®^ lots manufactured consecutively. The red line marks the maximum Ginkgolic acid concentration of 5 ppm allowed according to European pharmacopeias.

The proprietary *G. biloba* extract EGb 761^®^ involves a patented process (Figure 2). First, the cut plant material from all three harvest regions (US, China, France) is mixed to a unique blend, with the aim to achieve constant quality of the final product in spite of seasonal and regional variation of leaves in the respective harvest regions. This blending process for constant quality can be regarded analogous to wine blends referred as cuvée. The blend of *G. biloba* leaves is extracted by 60% (m/m) aqueous acetone. After separation of the primary extract from the exhausted plant material, acetone is removed by evaporation to leave an aqueous solution. This aqueous solution is cooled while stirring to induce a precipitation of chlorophyll, biflavones and the major bulk of ginkgolic acids, which represents the second waste stream. After removal of the precipitate, the residual aqueous solution is loaded with ammonium sulfate and extracted with a mixture of acetone and methylethylketone in a countercurrent fashion. While pure water and acetone are able to mix in any ratio, they effectively separate using highly kosmotropic salts like ammonium sulfate. The resulting aqueous phase represents another waste stream. The acetone-methylethylketone phase is highly concentrated by evaporation of the solvent and subsequently diluted with water. This resulting, mainly aqueous solution is extracted by butanol, however, in this case in a multiple static fashion, since butanol and water need a lot of time to separate thoroughly. The aqueous phase is the next waste stream, while the butanol phase is again highly concentrated by evaporation of the solvent and subsequently diluted with water. This resulting mainly aqueous solution is extracted with heptane, to remove essential oils and the last remaining traces of ginkgolic acids, and achieve the ≤5 ppm specification (Figure 3). The heptane phase is the last waste stream of the process, however, like the other organic solvents in the process, it is recycled continuously to minimize consumption of hydrocarbons. The mainly aqueous phase is concentrated by evaporation of the solvent and dried to yield the final extract.

Also a branch duct in the process at the stage of the acetone-methylethylketone can be applied, separating into a flavonol glycoside rich and a terpene lactone rich fraction which can be recirculated according to the desired concentrations in the final product (Waimer et al., 2016) (Figure 2 bottom).

**FIGURE 4:**
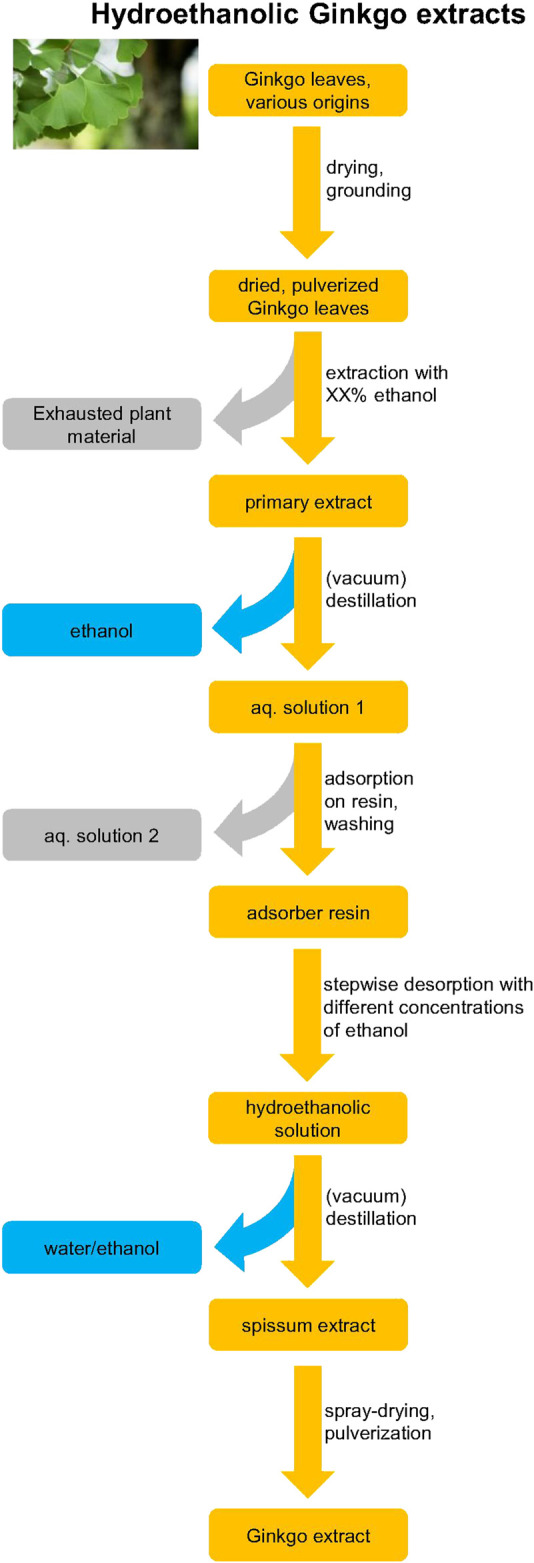
Scheme of the production of hydroethanolic *G. biloba* extracts according to, e.g., Chinese pharmacopoeia. The concentration of ethanol used for primary extraction may vary from 30 to 95% between manufacturers. Color coding: orange–value phase, gray–waste phase, blue–solvents. The hydroethanolic process differs to a major extent from the EGb 761^®^ process.

In contrast to the aforementioned process specific for EGb 761^®^, other manufacturers use a different order of the process steps. Moreover, the solvents used for the liquid-liquid extractions in these processes differ. First, in some cases hexane is substituted for heptane. Secondly, a mixture of toluene and butanol instead of pure butanol may be used. Thirdly, ethylacetate instead of a mixture of acetone with methylethylketone, without ammonium sulfate may be used (Giori et al., 2010) Also other details like temperatures, times, concentrations, etc. may be very different from the EGb 761^®^ process. Even more disparate processes (Figure 4) are mainly used by Chinese manufacturers, which carry out the primary extraction with a hydroethanolic mixture instead of a hydroacetonic mixture. Furthermore, powdered plant material is used instead of the cut material like in the EGb 761^®^ manufacturing. Subsequently, a resin adsorption and desorption as specified in the ChP is performed instead of the multistep process of liquid-liquid extractions. Although the final products of all these different processes share similar specifications on the flavonol glycosides, the residual phytochemical composition may vary, which is discussed in detail below. In conclusion, the combination of a unique blend of plant material of highest possible quality and a unique patented extraction process capable of adjusting concentrations of major ingredients results in a final product of unique composition with a very high batch-to batch consistency. Due to the multidimensionality of all these parameters, it is virtually impossible to produce an extract of the same composition by other manufacturers, which was already shown by targeted (Schötz et al., 2015) and untargeted principal component analyses (Kulić et al., 2021a).

Another issue is the occurrence of environmental pollutants, e.g., polycyclic aromatic hydrocarbons (PAH) and man-made chemicals, e.g., pesticides, in final products. PAHs are potent genotoxins frequently occurring in our environment (Alegbeleye et al., 2017). Some *G. biloba* -containing food supplements were shown to be heavily contaminated with these contaminants. For instance, of 66 *G. bilob*a leaf-containing food supplements, the maximum allowed level of 10 μg/kg benzo [a]-pyrene for food supplements with botanical ingredients was exceeded up to 6.4-fold and a combined value of four different PAH was even exceeded up to more than 10-fold (Martena et al., 2011).

Plant material used to produce EGb 761^®^ not harvested from own plantations is usually analyzed for PAH contamination before use. Moreover, every 10th extract lot is checked for such contaminations, in which undesirable deviations have never been found. Independent of that, a potential removal of PAH traces may be carried out using a patented extraction step (Hauer et al., 2006), which is, however, usually not necessary, since the plant material is free of detectable PAHs, as verified by the aforementioned analyses.

## The result generated by the process

The European pharmacopoeia requires that dried *G. biloba* leaf is to be extracted with 60% acetone (m/m) as primary extraction solvent and the final product is adjusted to 22.0%–27.0% ginkgo flavonoids calculated as ginkgo flavone glycosides and 5.4%–6.6% terpene lactones consisting of 2.8%–3.4% ginkgolides A, B, C, and 2.6%–3.2% bilobalide and contains less than 5 ppm ginkgolic acids (European Directorate for the Quality of Medicines & HealthCare, 2020). In contrast, the Chinese and US pharmacopoeias deviate in part from these specifications (Liu et al., 2021). While the US specification (United States Pharmacopeial Convention, 1979 ff.) for flavonol glycosides and ginkgolic acids are the same as the European, the specification for terpene trilactones can be 5.4%–12.0%. The Chinese pharmacopoeia specifies only lower limits of at least 6% terpene trilactones and at least 24% flavonol glycosides with no upper limits (Chinese Pharmacopoeia Commission, 2015).

In addition, in non-regulated markets, *G. biloba* extracts are not necessarily subject to proper quality controls and the standardization specified above is either not mandatory or simply ignored. A study of 27 *G. biloba* leaf extracts in the U.S. found that a majority did not even meet the specifications stated on the package insert (Kressmann et al., 2002b). The potentially harmful ginkgolic acids were present in concentrations of up to 9% instead of the 0.0005% permitted in Europe and in the US. A Dutch study showed similarly strong differences in quality among 29 ginkgo products (Fransen et al., 2010). Significant differences in quality between various *G. biloba* preparations were also found in a Japanese study (Kakigi et al., 2012). Among the preparations analyzed in a Polish study, products were found containing relatively small amounts of flavone glycosides or terpene lactones, but with ginkgolic acid concentrations of up to 392 ppm (Gawron-Gzella et al., 2010). In a British study, 33 of 35 examined *G. biloba* products were adulterated, or contained the desired compounds in only minor concentrations compared to EGb 761^®^ (Booker et al., 2016). Three of eight Gingko products purchased from Denmark and Australia were also adulterated (Wohlmuth et al., 2014). The analysis of 13 *G. biloba* leaf samples, 15 standardized powdered extracts, and 14 finished *G. biloba* products showed that only three of the finished products were not adulterated (Ma et al., 2016). When analyzing extracts or finished products, it is often not clear whether adulteration occurred at the drug level or at later stages of the production process. The vast peer-reviewed literature on *G. biloba* extract adulteration and measures to expose it has been reviewed elsewhere (Gafner, 2018; Gafner, 2022). In that context, the extract EGb 761^®^ has been called the “gold standard” in the literature by independent research groups, as it consistently met the specifications given by past and present pharmacopoeias (Wohlmuth et al., 2014).

Due to the requirements of the pharmacopoeias and scientific evidence from literature, the two compound classes that have attracted the most attention are the ginkgo-flavonol glycosides and the terpene trilactones (Isah, 2015; Nash and Shah, 2015; Schäfer, 2015; Feng et al., 2019; Achete de Souza et al., 2020; Sarkar et al., 2020). As explained above, European, US and Chinese Pharmacopoeias all specifically regulate the content of these compound classes in *G. biloba* leaf extracts. It is not entirely clear though, whether terpene lactones and flavonoids are that regulated because they have been that well characterized, or whether their regulation drew researcher’s attention particularly to these compound classes.

However, even two high-quality extracts complying with the same pharmacopoeia guideline can be disparate in nature. For instance, a range of 22%–27% flavone glycosides seems to be a relatively broad because if extract A contains 22% flavone glycosides and extract B contains 27%, extract B could contain almost 23% more of that compound class compared to extract A. But for herbal extracts with concomitant quantification levels for several distinct constituent classes, as is the case for *G. biloba* extracts with flavone glycosides, ginkgolides and bilobalide, meeting these multiple specifications throughout all batches is a challenge and requires excellent control of raw material sourcing and a sophisticated extraction process as stated above. Therefore, it is important to note that in spite of this multi compound adjustment the batch-to-batch variation is miniscule in EGb 761^®^, where variation of analyzed constituents is much lower compared to products from different manufacturers (Hui et al., 2015; Schötz et al., 2015).

Moreover, about 110 different flavonoids have been detected in *G. biloba* leaves (Liu et al., 2021). Thus, even two compounds with both 26% Ginkgo flavonoids can differ in their flavonoid composition. Among others, *G. biloba* extracts may contain the flavonoids myricetin, quercetin, apigenin, kaempferol, syringetin, and isorhamnetin, which may be glycosylated with various sugars such as rutinose, rhamnose or glucose (Wu et al., 2016; Yang et al., 2016). Thus, depending on the extraction method, e.g., polarity of the solvents used, the individual flavonoid glycosides could be present in different proportions. In order to detect potential adulterations, the peak area ratio of quercetin to kaempferol and isorhamnetin to quercetin are specified in Chinese pharmacopoeia to 0.8–1.2 and >0.15, respectively (Liu et al., 2021).

In addition, substances making up the unspecified approximately 70% may also contribute to efficacy, and may also be different between manufacturers, while remaining nearly constant in EGb 761^®^ (Biber, 2003; Kulić et al., 2021a). The mismatch between “the plethora of papers” on Ginkgo flavonoids and terpene lactones and the methodology to analyze those on the one hand and other compounds such as proanthocyanidins (PACs), shikimic acid, or 6-hydroxykynurenic acid has been bemoaned already quite a few years ago (van Beek, 2002; van Beek and Montoro, 2009). However, some early work had recognized the importance of PACs and the differences in their contents among different species (Schrall and Becker, 1977; Stafford et al., 1986). In addition, the difference in content and composition of organic acids was found to vary between EGb 761^®^ and Chinese *G. biloba* extracts: while EGb 761^®^ contains approximately 14% organic acids, with shikimic acid being the major compound, Chinese *G. biloba* extracts contain only around 2% organic acids, with shikimic acid being only a minor component among the residual portion of phenolic acids (Zhang et al., 2021).

The proanthocyanidins (PACs) have attracted attention as another major class of compounds in *G. biloba* leaves (Qa’dan et al., 2010), primary *G. biloba* extracts (Qa’dan et al., 2011), or EGb 761^®^, respectively. This class of compounds accounts for approximately 7% of EGb 761^®^, with a high batch-to-batch consistency (Kulić et al., 2021b). Due to the heterogenous production processes, the content of Ginkgo-PACs in products from different manufacturers can vary considerably. As will be discussed below, PACs can have profound pharmacologic effects and thus the amount in extracts should be evaluated.

As another general concept of plant extracts and plant-based medication, the active agent is the complete extract with all its constituents, and the combination of constituents is considered to have synergistic effects. On the other hand, it is in theory also conceivable that for specific indications, isolated compounds may be more suitable than the whole extract. For instance, alkaloids are known to be a highly potent class of natural products, which are often the sole pharmacophores within the respective extracts. Thus, alkaloids are more effective when applied as pure molecules without the “impurities” of the residual extract bulk. However, for alkaloid-free plant extracts, which are mainly composed of polyphenols, organic acids and terpenes, apart from few exceptions like e.g. cardiac glycosides these considerations usually do not apply, and the aforementioned synergistic concept is the predominant active principle. For Ginkgo extracts and the respective constituents, some studies support these principles for different indications, respectively. For instance, in a pilot study involving 104 patients, not testing whole *G. biloba* extract, but Ginkgolide B at 240 mg or 360 mg/d or placebo over only 7 days, a trend for improvement in multiple sclerosis (MS) patients was observed compared to placebo (Brochet et al., 1995).

For the treatment of migraine, clinical results describing an efficacy of ginkgo extract constituents have been described, for example to prevent migraine in adolescents 8–18 years using 2 × 80 mg ginkgolide B over 3 months (Usai et al., 2010; Usai et al., 2011); for prophylaxis in women with migraine with aura using 2 × 60 mg gingko terpenes for 3 months (D'Andrea et al., 2009); for the treatment of acute migraine with aura in men and women using 2 x 60 mg/d ginkgo terpenes at the onset of the migraine attack (Allais et al., 2013). As those examples suggest, in principle it is possible that additional single *G. biloba* constituents will be developed into drugs for specific disease indication areas.

## Highest quality: Is it worth all the trouble?

So far, we have discussed the product-by-process concept, i.e., the fact that procedures from the tree care up to the extraction process affect the composition of the final medicinal product. It has been pointed out that the “gold standard” (Wohlmuth et al., 2014), “the most widely studied extract, EGb 761^®^” (Gafner, 2022) differs from other dry extracts on the market (Gafner, 2018; Gafner, 2022).

Thus, the differences between various extracts are not disputable. However, does the quality of the final product really matter? Does it affect efficacy and safety? In this chapter, we will first look at arguments why it seems plausible that the effort in generating consistently high-quality extracts of uniform composition is vital for efficacy and safety (chapter “Intuitively plausible: Why would different extracts act differently?”). Although head-to-head comparisons of the effects and the tolerability of different extracts made from the same plant species are scarce, we will next present the instances in which those differences have been demonstrated, suggesting consequences of the final product constitution for the user (chapter “Appreciation of the individual: Different extracts display different effects”). These points have been considered by experts, and many local and international guidelines as well as review articles clearly distinguish between different *G. biloba* extracts, which we will briefly discuss in chapter “Common sense, but common knowledge not yet: Appreciation of the product by process in the literature”. Last but not least, we will briefly provide some information on the overwhelming vastness of the evidence of efficacy generated using EGb 761^®^ in a few selected indication areas.

### Intuitively plausible: Why would different extracts act differently?

As explained above, much research has been conducted on those compounds used to “standardize” *G. bilob*a extracts, i.e., the flavonoids and the terpene lactones. As explained above, even those may differ between different extracts that formally fulfil the pharmacopoeia specifications. Extracts produced using dissimilar methodology may vary with respect to compounds that are not regulated by pharmacopoeias, and that have attracted relatively little attention so far. Although research on those compounds is at its fledgling stages, there is quite some profound evidence about their pharmacologic effects.

For instance, the aforementioned *G. biloba* PACs are a group of compounds that are plausible to contribute to pharmacologic effects, as shown for PACs of other sources: In people >58 years, the risk of developing dementia at an average follow-up of 6.7 years was substantially reduced (HR = 0.69, 95% CI 0.48, 0.99) in people with high dietary PAC intake compared to those with low PAC intake (Agarwal et al., 2019).

How about PACs from *G. biloba* leaf extract? Some data regarding PACs derived from *G. biloba* extracts have to be interpreted with caution. For instance, some of the catechins and procyanidins in *G. biloba* leaf extract were identified as (+)-catechin, (−)-epicatechin, (−)-gallocatechin, (−)-epigallocatechin and procyanidins B1 and B3 (Xie et al., 2014). *In vitro*, all six compounds potently inhibited Aβ42 aggregation and destabilized preformed fibrils, i.e., they interfered with a process associated with Alzheimer’s disease (Xie et al., 2014). However, enticing as those results may seem, the total contents of those monomeric and dimeric compounds were determined only in minute amounts (ppm scale), so that the activities described in that study are unlikely to provide relevant contribution for the pharmacologic effects observed in *G. biloba* leaf extracts. However, other work described here was done using the higher oligomeric PACs, which are present in concentrations more likely to contribute to the overall pharmacologic effects of the extract. For instance, Ginkgo PACs were found to be potent radical scavengers *in vitro* in cell-free systems (Qa’dan et al., 2011; Cao et al., 2018a; Ellnain-Wojtaszek et al., 2003), in an assay using retinal pigment epithelial cells (Li et al., 2021), or in a neuronal cell line (Sens-Albert et al., 2021a). Interestingly, medication containing EGb 761^®^ had a more potent anti-oxidative effect than medication containing *G. biloba* extract with a much lower PAC content. That result is consistent with the assumption that PACs actually contribute to pharmacologically relevant effects of the whole *G. biloba* extract.

In a rat cardiac injury model, PACs and *G. biloba* extract exerted their cardioprotective effects *via* similar, anti-oxidant and anti-apoptotic effects, which is again consistent with the assumption that the PACs in *G. biloba* extract contribute to those desirable effects (Boghdady, 2013).

However, PAC effects go beyond mere anti-oxidative properties. *G. biloba* PACs may inhibit human chymase, a protein involved in inflammatory processes, hypertension, and arteriosclerosis (Dubey et al., 2016).

In a rat ischemia model, i. p. applied *G. biloba* PACs effectively mitigate behavioral defects, decrease infarct volume, increase superoxide dismutase activity in the brain, and decrease malondialdehyde and nitric oxide brain concentrations (Cao et al., 2016; Yao et al., 2020). The PAC fraction isolated from EGb 761^®^ reduced scopolamine-induced impairment of short-term memory in mice (Sens-Albert et al., 2021b), suggesting *in vivo* pharmacological activity of this compound group upon oral application.

In addition to PACs, pharmacological effects of 6-hydroxy kynurenic acid (6-HKA) have been demonstrated. *In vitro* studies showed antagonistic activity of 6-HKA against the AMPA and NMDA receptors (Weber et al., 2001). In a rat model oral administration of 6-HKA isolated from a *G. biloba* leaf extract at a dose of 10 mg/kg provided partial protection against cerebral ischemia and reperfusion injury. Albeit the overall effect was less pronounced than that of the Ginkgo-PAC fraction, Yao et al. (2020) suggest that 6-HKA potentially contributes to the overall efficacy of *G. biloba* extracts. However, this conclusion also needs to be taken with caution, since 6-HKA is a substance present in sub-% concentrations in most *G. biloba* extracts.

Shikimic acid is another quantitatively relevant constituent in EGb 761^®^, which was, however, shown to be present in a 40-fold lower concentration in *G. biloba* extracts manufactured by the hydroethanolic process (Zhang et al., 2021). Shikimic acid has been shown to exert protective activity against oxidative stress *in vitro* in human neuroblastoma cells (Rabelo et al., 2015). In a macrophage cell line, shikimic acid suppressed the LPS-induced upregulation of inflammatory indicators (Rabelo et al., 2016). In an inflammation mouse model, it decreased nociception. Those results are particularly relevant, because many of the effects observed in patients are attributed to the anti-inflammatory activity exerted by *G. biloba* extracts (For recent reviews, see E-Tabassum et al., 2022; Mousavi et al., 2022; Al-Kuraishy et al., 2022; Barbalho et al., 2022; Ibrahim et al., 2021). Moreover, shikimic acid positively affected oligodendrocyte precursor differentiation *in vitro* and improved remyelination in a mouse model of experimental autoimmune encephalomyelitis *in vivo*, suggesting at least some extent of pharmacological activity in the CNS (Lu et al., 2019). Concerning the established *G. biloba* indications, shikimic acid administration reduced focal cerebral ischemia injury in a rat model of middle cerebral artery thrombosis (Ma et al., 1999). To date there is no direct evaluation of the relevance of this constituent to the efficacy of *G. biloba* extracts. However, given its relevant and strongly varying content in *G. biloba* extracts from different manufacturing processes (Zhang et al., 2021) and the demonstrated pharmacological activity profile, differences in shikimic acid content could potentially affect the efficacy profile of different *G. biloba* extracts.

In summary, as the compound concentrations between different *G. biloba* extracts substantially differ, and as those compounds exert pharmacologic effects, it is plausible to surmise that different extracts–even when coming from the same plant-can have different pharmacologic properties. In the following chapter, we will discuss more direct confirmations of that assumption.

### Appreciation of the individual: Different extracts display different effects

Using a sophisticated multi-electrode array to examine connectivity and many other parameters on primary cultures of mouse cortical neurons, the effect of various *G. biloba* medications purchased from a pharmacy were used in a model for protection against toxic Aβ42 (Bader et al., 2018). In this “brain on a chip” model, medication containing differently prepared extracts displayed different effects. Medication containing EGb 761^®^ was the most potent protector of interneuronal signal transmission and nerve cell networking against Aβ42, compared to all other 5 *G. biloba* preparations tested. The study confirms that different medication adhering to the very same pharmacopoeia specifications can profoundly differ in their effects in relevant models for neurodegenerative disease.

Although the model used by Bader et al. (2018) replicates many features of the whole brain, it cannot supersede *in vivo* experiments. Comparing two finished products in a mouse model of spontaneous alternations in the T-maze, the EGb 761^®^ containing product more efficiently protected against scopolamine-induced cognitive impairment than another product containing *G. biloba* extract with a lower PAC content (Sens-Albert et al., 2021a; Sens-Albert et al., 2021b). Those observations verify the assumption that EGb 761^®^ and other extracts are dissimilar in their effects both *in vitro* and *in vivo*.

The aforementioned experiments were conducted in preclinical models. To examine whether disparate effects of various *G. biloba* preparations could be observed in human, the effects of three different commercially available *G. biloba* products, including one containing EGb 761^®^ (Ginkgold^®^) were examined (Itil and Martorano, 1995). All preparations contained 24% Ginkgo flavone glycosides. In 12 healthy male volunteers, quantitative pharmaco EEGs were obtained in a double-blind crossover method of investigation. The EGb 761®-containing medication increased alpha activity in all brain areas, which was not observed to that extent with the other *G. biloba* preparations. Overall, the activation pattern by Ginkgold could be correlated with the activation pattern of other known cognitive enhancers, which was not the case for the other two preparations. These data substantiate the different effects observed in preclinical models also in human.

The latter experiments have been conducted in healthy people, rather than patients. We are not aware of head-to-head comparisons of different extracts in patients. Thus, we are reduced to conjectures, that are, however, quite compelling.

In another review study it was found that in each one of eight clinical trials with a total of 1,199 patients a positive effect was demonstrated for treating tinnitus by EGb 761® compared to placebo (von Boetticher, 2011). A more recent meta-analysis of clinical studies examining efficacy of EGb 761® to treat tinnitus in dementia patients also found a clear and significant effect of EGb 761® compared to placebo (Spiegel et al., 2018). That effect is mediated in part by EGb 761® improving cognition, anxiety, and depression, which in turn alleviates tinnitus (Brüggemann et al., 2021). These positive data appear to be at odds with the results from a large clinical trial involving 1,121 patients in which such efficacy in tinnitus treatment could not be demonstrated (Drew and Davies, 2001). It is not clear whether that difference was due to the fact that this work was conducted using a *G. biloba* extract different from EGb 761®. Many methodological shortcomings of the trial design also need to be taken into account.

Interestingly, two finished products can display different pharmacokinetics regarding constituents that are present at the same concentrations in both preparations. In a single dose crossover design, 12 volunteers were treated with either 120 mg EGb 761^®^ containing tablets or with capsules containing another standardized extract, with both containing similar amounts of terpene lactones (Kressmann et al., 2002a). Compared to EGb 761^®^, the bioavailability of the other *G. biloba* extract was dramatically lower, which may have been due to a lower bioavailability of the final product compared to EGb 761®-containing tablet. This result implies, that the galenic formulation is of major importance for the bioavailability of the effective constituents of *G. biloba* extracts.

### Common sense, but common knowledge not yet: Appreciation of the product by process in the literature

Considering that even coffee tastes different and contains divergent constituents when it has been brewed using different methods, the fact that different procedures render different products may appear quite intuitive. As a corollary of what we have discussed so far, there can hardly be any true “generics” of plant-based extracts, which has been pointed out in pharmaceutical guidelines: “In the case of herbal medicinal products, the complexly composed extract is considered the active ingredient. Starting from a certain medicinal raw material, very different extracts can be produced depending on the extraction agent and process. Therefore, for the decision whether an herbal medicinal product can be substituted by another medicinal product it is not sufficient to ensure that both products are based on the same drug. Extracts that differ in one of the declared parameters cannot be considered as “identical to the active substance” If herbal medicinal products are declared identical in the specification characteristics mentioned, they may be preparations also identical in active substance. However, this is not necessarily the case. In addition to these parameters, active ingredients with such complex compositions as herbal extracts have a large number of other characteristics that may vary if the extracts were not manufactured using an identical processes. Ultimately, one can only be certain that two preparations contain identical - and thus interchangeable extracts if a “development name” (e.g., EGb..., LI..., WS... etc.) assigned to the extract is given in the expert information or in another reliable source of information, indicating that it was manufactured using identical processes. The general principle that modified dosage forms are not interchangeable also applies to herbal medicinal products.” (translated from Blume et al., 2014).

In the current German guideline for dementia treatment, specifically *G. biloba* extract EGb 761^®^, but no other *G. biloba* preparations, is recommended as a possible treatment for mild to moderate vascular or Alzheimer’s dementia with the highest evidence level 1 a (Deuschl et al., 2016). Likewise, guidelines of the World Federation of Societies of Biological Psychiatry (Ihl et al., 2015) and the Swiss guidelines for the diagnostics and treatment of behavioral and psychological symptoms of dementia (Savaskan et al., 2014) specify the evidence-based *G. biloba* extract as EGb 761^®^. In a review examining international clinical guidelines, it was found that EGb 761^®^ was the only Ginkgo-based pharmacological treatment recommended in clinical guidelines (Kasper et al., 2020).

In many review articles, specifically EGb 761^®^, rather than “Ginkgo extract” was examined, or the authors carefully distinguished between different extracts (for a few selected examples, see Gauthier and Schlaefke, 2014; Barth et al., 2021; DeFeudis, 1991; Spiegel et al., 2018; Tomino et al., 2021; Zhang et al., 2016; Riepe, 2020; Farzaei et al., 2017; Müller et al., 2019; Hashiguchi et al., 2015; Solfrizzi and Panza, 2014; Tan et al., 2015). In other studies, the distinction was less stringent or not implemented at all (for a few selected examples, see Al-Kuraishy et al., 2022; Dutta et al., 2022; Zhao et al., 2021; Hallak et al., 2021; Ibrahim et al., 2021). Thus, it may be misleading to generalize evidence gained for one specific extract to all products made from the same plant. Such imprecision violates the postulate of scientific meticulousness and may even lead to disease treatment decisions that are not supported by objective evidence.

## Conclusion

While little is known about the effects and tolerability of most *G. biloba* extracts, much data and evidence is available for EGb 761^®^. A PubMed search for EGb 761^®^ renders over 2000 articles (as of July 2022), and EGb 761^®^ has been called “the most widely studied extract in clinical research” (Martinez-Solis et al., 2019). That is one of the reasons we selected EGb 761^®^ as a paradigm to illustrate the product-by-process concept.

However, what has been scrutinized here does not only apply to *G. biloba*, but to other plant extracts as well. What is commonplace to the vintner, applies even more to the science of phytopharmacology: All beverages sold under the label “wine” should fulfil a modicum of characteristics regarding its manufacturing–which is analogous to the European pharmacopoeia regulations for Ginkgo extract–but not all wines fulfilling legislative standards are equal. The product-by-process concept should become even more deeply incorporated in the reasoning of scientists, physicians, pharmacists and the educated lay public.

## Abbreviations

6-HKA, 6-hydroxykynurenic acid; ChP, Chinese Pharmacopoeia; DER, Drug (dried Plant material) to Extract Ratio; PAH, Polycyclic aromatic hydrocarbons; Ph. Eur, European Pharmacopoeia; USP, US Pharmacopoeia; PAC, Proanthocyanidin.
